# Prophylactic PEG-rhG-CSF Reduces Febrile Neutropenia in Pediatric Hematological Malignancies Compared with Daily rhG-CSF

**DOI:** 10.3390/cancers18142214

**Published:** 2026-07-09

**Authors:** Xiao Zhang, Yang Fu, Hongsheng Wang, Xiaohua Zhu, Yi Yu, Ping Cao, Chen Shen, Xiaowen Zhai

**Affiliations:** Department of Hematology, National Children’s Medical Center, Children’s Hospital of Fudan University, Shanghai 201102, China

**Keywords:** child, efficacy, hematological malignancies, pegylated recombinant human granulocyte colony-stimulating factor, safety

## Abstract

**Background**: Febrile neutropenia (FN) is a major complication of chemotherapy in pediatric hematological malignancies. This study compared the efficacy and safety of prophylactic pegylated recombinant human granulocyte colony-stimulating factor (PEG-rhG-CSF) versus daily short-acting recombinant human granulocyte colony-stimulating factor (rhG-CSF). **Methods**: This single-center, open-label, randomized controlled trial was conducted at a tertiary children’s hospital in China. Pediatric patients (<18 years) with confirmed hematological malignancies (leukemia or lymphoma) were enrolled. A total of 138 chemotherapy cycles were randomized 2:1 to receive either a single dose of PEG-rhG-CSF (100 μg/kg, *n* = 86 cycles) or daily short-acting rhG-CSF (5 μg/kg/day, *n* = 45 cycles) after chemotherapy. The primary endpoint was FN incidence. Secondary endpoints included neutropenia incidence, FN duration, time to neutrophil recovery, blood product transfusions, hospital stay, and costs. Safety was assessed by monitoring adverse events (AEs) graded according to NCI CTCAE version 4.03. **Results**: Baseline characteristics were comparable between groups. PEG-rhG-CSF significantly reduced the incidence of FN (59.3% vs. 77.7%, OR 0.42 (0.18–0.97), *p* = 0.046) compared to daily rhG-CSF. The median time to neutrophil recovery was 9.8 days (95% CI: 8.1–10.7) in the PEG-rhG-CSF group and 10.3 days (95% CI: 8.6–11.1) in the control group (*p* = 0.45). No significant differences were observed in FN duration, transfusions, hospital stay, or costs. Subgroup analyses showed PEG-rhG-CSF significantly reduced FN in non-Hodgkin lymphoma (57.4% vs. 83.3%, OR 0.27 (0.08–0.89), *p* = 0.032). A total of 12 AEs (9.9% overall, mainly bone pain) were observed, with no significant difference between groups and no infection-related deaths. **Conclusions**: In this single-center, open-label trial, prophylactic single-dose PEG-rhG-CSF was associated with a lower incidence of FN compared to daily short-acting rhG-CSF. Safety profiles appeared broadly similar, but the sample size was insufficient to detect rare differences.

## Simple Summary

Chemotherapy can cause a serious side effect called febrile neutropenia (fever with low white blood cells) in children with blood cancers, which may lead to infections and treatment delays. Daily growth factor injections are often used to prevent this, but they are burdensome for young patients and their families. This study compared a single injection of a long-acting growth factor (PEG-rhG-CSF) with daily standard injections in children receiving chemotherapy for leukemia or lymphoma. The results showed that the single injection reduced the occurrence of low white blood cell counts and fever compared to daily injections, with similar safety. A single dose per chemotherapy cycle is more convenient and may improve the quality of life for children and their caregivers. These findings support the use of single-dose PEG-rhG-CSF as an effective and practical option for preventing chemotherapy-related complications in pediatric blood cancers.

## 1. Introduction

Febrile neutropenia (FN) is most commonly observed in patients receiving cytotoxic chemotherapy, affecting 10–50% of patients with solid tumors and over 80% of those with hematological malignancies. Its complications include treatment delays, chemotherapy dose reductions, and an increased risk of infection [1,2,3].

For pediatric patients at high risk of FN, besides prophylactic empirical antibacterial therapy, primary and secondary prophylaxis with recombinant human granulocyte colony-stimulating factor (rhG-CSF) represents a key management strategy [4]. However, a significant limitation of rhG-CSF is its short half-life, necessitating daily administration. Pegylation of rhG-CSF (PEG-rhG-CSF) reduces renal clearance and prolongs its circulating half-life, thereby sustaining drug activity over an extended period [5]. Nevertheless, due to the particularities of the pediatric population, research on the use of long-acting G-CSF in children with hematological malignancies remains relatively scarce. Currently, there is only one guideline addressing FN management in pediatric and adolescent cancer patients [4], and a recent meta-analysis on the impact of PEG-rhG-CSF on FN incidence in pediatric cancer patients was limited by the small number of available studies and substantial heterogeneity [6].

Therefore, this study aimed to compare the efficacy and safety of prophylactic PEG-rhG-CSF versus short-acting rhG-CSF in children with hematological malignancies after chemotherapy, to provide clinical evidence for FN prevention and management in this population.

## 2. Methods

### 2.1. Study Design and Participants

This single-center, prospective, open-label, randomized controlled trial was conducted at a tertiary children’s hospital in China. Eligible participants were children (<18 years) with a confirmed diagnosis of pediatric hematological malignancy (leukemia or lymphoma), scheduled to receive prophylactic G-CSF after chemotherapy with a minimum 12-day interval between cycles. Key exclusion criteria included severe organ dysfunction, allergy to study drugs, or participation in another clinical trial within 4 weeks. This study was registered and filed with the National Health Commission of the People’s Republic of China through the National Medical Research Registration and Filing Information System (Filing code: 9450100201000RQ). The registration was completed prior to the enrollment of the first participant, with the date of registration being 2 December 2020. As an investigator-initiated trial not intended for drug registration, this study was conducted in accordance with the requirements of the National Health Commission for the management of medical research projects. Written informed consent was obtained from all legal guardians. CONSORT for randomized clinical trials see File S1.

### 2.2. Randomization and Procedures

Eligible chemotherapy cycles were randomly assigned in a 2:1 ratio to receive either PEG-rhG-CSF or short-acting rhG-CSF using sequentially numbered, opaque, sealed envelopes. Each eligible chemotherapy cycle from the same patient was independently randomized, meaning that a patient contributing multiple cycles could receive PEG-rhG-CSF in one cycle and short-acting rhG-CSF in another, depending on the randomization result for that specific cycle. The experimental group received a single subcutaneous injection of PEG-rhG-CSF (100 μg/kg; CSPC Pharmaceutical Group Limited, Shijiazhuang, China) 24–72 h after chemotherapy. The control group received daily subcutaneous injections of short-acting rhG-CSF (5 μg/kg/day) starting 24–72 h post-chemotherapy until the absolute neutrophil count (ANC) recovered to >0.5 × 10^9^/L for two consecutive days. Chemotherapy regimens were administered according to standard protocols (see Supplementary Table S1 and File S2).

### 2.3. Outcomes

The primary efficacy endpoint was the incidence of FN, defined as ANC < 0.5 × 10^9^/L accompanied by a single oral temperature ≥ 38.3 °C or ≥38.0 °C for over one hour. Secondary endpoints included incidence of neutropenia (ANC < 0.5 × 10^9^/L), duration of FN, time to neutrophil recovery, number of blood product transfusions, length of hospital stay, and hospitalization costs. Safety was assessed by monitoring adverse events (AEs), graded according to NCI CTCAE version 4.03, for 30 days after treatment.

Hospitalization costs were assessed as a secondary endpoint. Hospitalization costs were defined as the total medical expenses incurred during each inpatient stay. These included general treatment service fees, surgical and non-surgical treatment fees, laboratory diagnostic fees, imaging diagnostic fees, Western medicine costs, antimicrobial drug costs, blood product costs, and the acquisition costs of the study drugs (both PEG-rhG-CSF and short-acting rhG-CSF).

### 2.4. Statistical Analysis

Categorical variables were compared using the Chi-square or Fisher’s exact test, and continuous variables using the independent samples *t*-test or Mann–Whitney U test, as appropriate, for descriptive baseline comparisons. For efficacy analyses, to account for within-patient correlation arising from multiple chemotherapy cycles per patient, binary outcomes (neutropenia and FN) were analyzed using generalized estimating equations (GEE) with an exchangeable working correlation matrix, reporting odds ratios (ORs) with 95% confidence intervals (CIs). Continuous outcomes were analyzed using linear mixed-effects models with patient ID as a random intercept, reporting mean differences (MDs) with 95% CIs. The GEE and mixed-effects models included treatment group as the fixed effect and patient ID as the cluster variable (GEE) or random intercept (mixed-effects models). No additional covariates were adjusted for in the primary analyses, as the randomized groups were well balanced at baseline. Time to neutrophil recovery was analyzed using the Kaplan–Meier method, and differences between groups were compared using the log-rank test; results are reported as medians with 95% CIs. All statistical tests were two-sided, and a *p*-value < 0.05 was considered statistically significant. Statistical analyses were performed using SPSS version 23.0 (IBM Corp., Armonk, NY, USA).

## 3. Results

### 3.1. Patient Disposition and Baseline Characteristics

**Figure 1:**
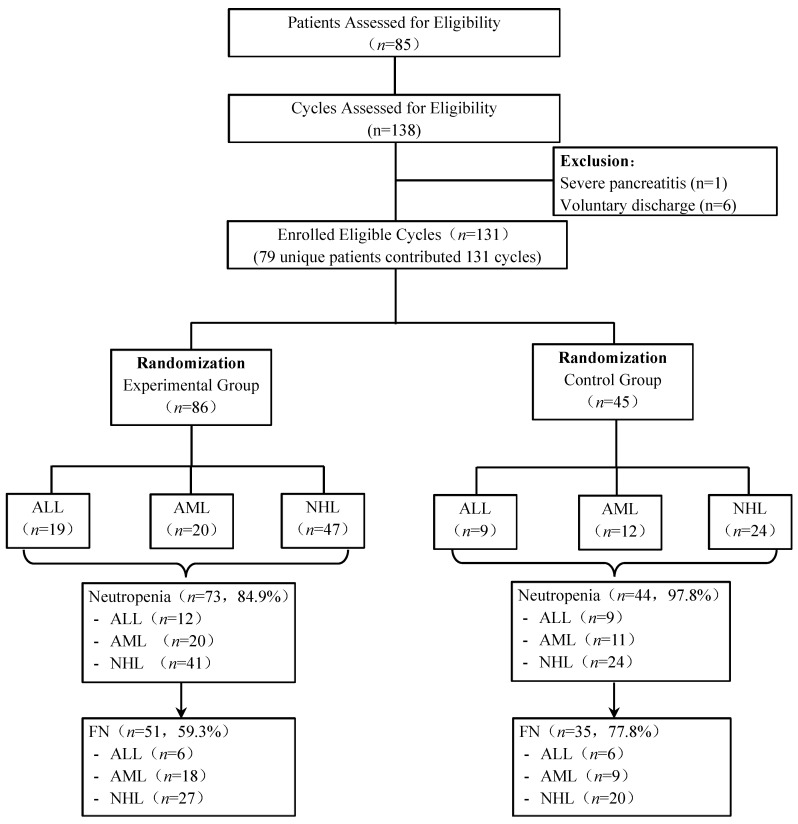
Study flow diagram. A total of 85 unique patients were initially assessed for eligibility. Among them, 79 unique patients were enrolled and contributed 131 chemotherapy cycles to the final analysis. Cycles were randomized in a 2:1 ratio to receive either PEG-rhG-CSF (experimental group, *n* = 86 cycles) or short-acting rhG-CSF (control group, *n* = 45 cycles). Exclusions included: 1 patient with severe pancreatitis during chemotherapy and 5 patients who voluntarily withdrew from the study. Randomization was performed at the chemotherapy-cycle level; therefore, some patients contributed multiple cycles to the analysis (mean 1.66 cycles per patient; range: 1–4 cycles). Disease subtype distributions are shown within each group.

A total of 138 chemotherapy cycles were enrolled from 85 unique patients who were initially assessed. After exclusions (see Figure 1), 131 chemotherapy cycles from 79 unique patients were included in the final analysis: 86 cycles from 53 unique patients in the experimental group and 45 cycles from 26 unique patients in the control group. The mean number of cycles per patient was 1.66 (range: 1–4 cycles). The ratio of experimental to control group cycles was 1.94:1 (Figure 1).

In the experimental group, 37 patients (69.8%) were male, with a mean age of 7.0 ± 3.8 years. In the control group, 16 patients (61.5%) were male, with a mean age of 6.7 ± 3.5 years. The most common primary disease was non-Hodgkin lymphoma in both groups (47 cycles, 54.7% in the experimental group vs. 24 cycles, 53.3% in the control group). The two groups were well balanced with respect to age, sex, disease type, chemotherapy phase distribution, and number of prior chemotherapy cycles (4.37 ± 3.28 vs. 4.87 ± 3.30), indicating that the randomization produced comparable groups (Table 1).

### 3.2. Efficacy Outcomes

**Figure 2:**
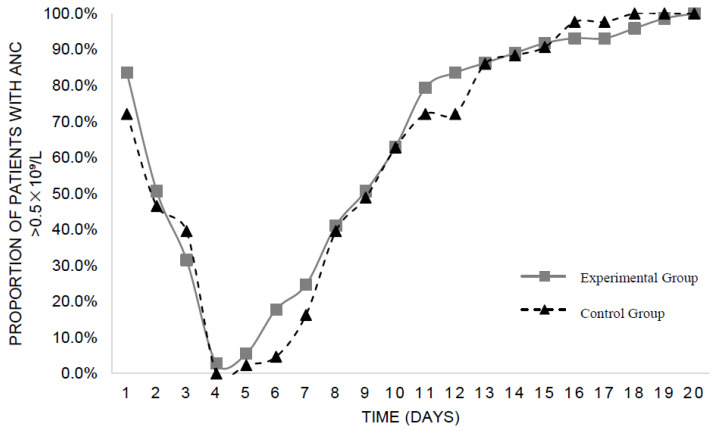
Comparison of time to neutrophil recovery between the PEG-rhG-CSF and short-acting rhG-CSF groups. Kaplan–Meier curves showing the proportion of patients achieving neutrophil recovery (absolute neutrophil count [ANC] > 0.5 × 10^9^/L) over time from the first dose of granulocyte colony-stimulating factor (G-CSF). The experimental group (solid line with square markers) received a single subcutaneous injection of pegylated recombinant human G-CSF (PEG-rhG-CSF, 100 μg/kg), while the control group (dashed line with triangle markers) received daily subcutaneous injections of short-acting rhG-CSF (5 μg/kg/day). Censored patients are indicated by vertical tick marks. The median time to neutrophil recovery was 9.8 days (95% CI: 8.1–10.7 days) in the PEG-rhG-CSF group and 10.3 days (95% CI: 8.6–11.1 days) in the control group, with no statistically significant difference between the groups (log-rank test, *p* = 0.45).

All reported *p*-values and odds ratios for binary outcomes (neutropenia and FN) were derived from GEE analyses, with patient ID as the cluster variable to account for within-patient correlation. The PEG-rhG-CSF group demonstrated a significantly lower incidence of FN (59.3% vs. 77.7%, OR 0.42 (0.18–0.97), *p* = 0.046) compared to the control group.The incidence of neutropenia was also lower in the PEG-rhG-CSF group (84.9% vs. 97.8%), although the difference did not reach statistical significance after adjusting for within-patient correlation (OR = 0.26, 95% CI: 0.05–1.26, *p* = 0.095). The median time to neutrophil recovery was 9.8 days (95% CI: 8.1–10.7) in the PEG-rhG-CSF group and 10.3 days (95% CI: 8.6–11.1) in the control group (*p* = 0.45). No significant differences were found between the groups in FN duration, number of transfusions, length of hospital stay, or hospitalization costs (Table 2; Figure 2).

Subgroup analysis by disease type revealed that PEG-rhG-CSF significantly reduced FN in the non-Hodgkin lymphoma subgroup (57.4% vs. 83.3%, OR 0.27 (0.08–0.89), *p* = 0.032) (Table 3). All subgroup analyses were post hoc and exploratory, and the small sample sizes limit the reliability of these findings. Interaction testing was not performed because the study was not powered for subgroup interaction analyses. These results should be interpreted with caution and considered hypothesis-generating.

### 3.3. Adverse Events

A total of 12 AEs (9.9%) attributed to G-CSF were observed, including bone pain (4 cases, 3.1%; 3 in experimental, 1 in control), rash (2 cases, 1.5%; 1 in each group, both improved after anti-allergy treatment), diarrhea (1 case, 0.8%; in control group), elevated liver enzymes (3 cases, 2.3%; 2 in experimental, 1 in control, all normalized within 2 weeks during follow-up), and chemotherapy delay (2 cases, 1.5%; 1 in each group). No infection-related deaths occurred in either group. The incidence of AEs showed no statistically significant difference between the two groups.

## 4. Discussion

FN is one of the most common complications and reasons for hospitalization in children receiving chemotherapy. Severe infections occur in 10–29% of FN episodes in pediatric cancer patients, with FN-related infection mortality ranging from 0.25% to 0.75% [7,8,9]. Cancer type (hematological vs. solid tumor) is significantly associated with the risk of severe infection, likely due to chemotherapy regimen types and dose intensity, leading to a higher incidence of infectious complications in hematological malignancies compared to solid tumors. A prospective study analyzing 1197 FN episodes (mean age 8 years) in two French pediatric cancer centers found that 66% of FN episodes occurred in children with hematological malignancies. The proportion of FN episodes with severe infection was 27% (129/481) in ALL, 38% (62/163) in AML, and 23% (32/141) in lymphoma [10].

Randomized controlled trials in both adults and children [11,12,13,14,15,16,17] have demonstrated that rhG-CSF prophylaxis for chemotherapy-induced neutropenia can reduce the incidence of neutropenia and FN, shorten the duration of neutropenia, decrease the frequency and duration of hospitalization, reduce antibiotic use, and improve outcomes in cancer patients. rhG-CSF has become a standard supportive care medication in pediatric hematological oncology [4]. For patients expected to experience prolonged neutropenia, rhG-CSF support can be used as an effective strategy during intensive therapy to prevent hematological toxicity and maintain dose intensity. Typically administered for about 10 days, this daily regimen can be burdensome for pediatric patients and their families. Even if parents learn self-injection, administering injections to young children often requires the assistance of at least two parents, potentially causing psychological and lifestyle burdens for the family and affecting the child’s quality of life.

PEG-rhG-CSF is formed by the selective conjugation of a 20 kDa polyethylene glycol molecule to the N-terminus of the rhG-CSF protein. The clearance of PEG-rhG-CSF is primarily mediated by neutrophils; thus, drug concentrations are maintained during chemotherapy-induced neutropenia and rapidly eliminated upon neutrophil recovery [18]. Studies in adults have shown that administration once per chemotherapy cycle is sufficient for effective prevention of neutropenia [19]. PEG-rhG-CSF has also demonstrated good efficacy and safety, improving overall survival in patients receiving chemotherapy [20,21,22,23,24]. Compared to rhG-CSF, it reduces the incidence of FN [25], with long-acting G-CSF outperforming short-acting G-CSF [26]. However, data on the efficacy and safety of PEG-rhG-CSF in children are limited.

Our study demonstrated that in pediatric patients with hematological malignancies receiving high-dose cytotoxic chemotherapy, prophylactic single-dose PEG-rhG-CSF (100 μg/kg) resulted in a lower incidence of neutropenia (84.9% vs. 97.8%) and FN (59.3% vs. 77.7%) compared to daily short-acting G-CSF (5 μg/kg/day). Compared to other international studies in pediatric oncology, the incidence of neutropenia in our study was relatively high, which might be related to the inclusion of chemotherapy phases containing high-dose cytotoxic drugs. Regarding the efficacy comparison between long-acting and short-acting G-CSF, a randomized controlled trial in pediatric sarcoma patients [27] reported similar efficacy and safety endpoints for PEG-rhG-CSF and rhG-CSF. Two other studies [28,29] also reported median durations of neutropenia of 5.0 days for PEG-rhG-CSF versus 6.0 days for rhG-CSF in pediatric patients receiving VDC/IE chemotherapy, a difference considered clinically insignificant. Regarding the potential confounding effect of G-CSF-containing AML regimens, we note that these regimens were distributed similarly between the two groups, and the G-CSF in these protocols was used for post-chemotherapy support, which is consistent with the prophylactic use in both study arms. Nevertheless, we acknowledge this as a potential confounder.

However, a meta-analysis in adults [30] indicated that PEG-rhG-CSF demonstrated superior efficacy and effectiveness compared to rhG-CSF in most studies, including lower incidences of neutropenia, FN, hospitalization, antibiotic use, and adverse events. In our study, PEG-rhG-CSF was superior to rhG-CSF in reducing the incidence of neutropenia and FN, but no statistically significant differences were found in other indicators, including time to neutrophil recovery, duration of FN, number of transfusions, length of hospital stay, and hospitalization costs. This might be because the pediatric hematopoietic system is more active than that of adults, with differences in hematopoietic stem cell composition and their niche [31]. Therefore, future study designs should consider stratifying children and adults to explore the effects of PEG-rhG-CSF versus rhG-CSF. The comparable hospitalization costs between the two groups may be explained by the fact that, although the unit price of a single PEG-rhG-CSF dose is higher than that of daily short-acting rhG-CSF, this cost difference was offset by the reduced frequency of administration, nursing time and injection-related consumables in the PEG-rhG-CSF group. It should be noted that this interpretation is based on a reasonable assumption rather than formal measurement of nursing time or consumable costs, which were not collected as secondary endpoints. Future cost-effectiveness analyses incorporating detailed breakdowns of drug and nursing costs are warranted.

In this study, a total of 12 AEs attributed to G-CSF were observed, including bone pain, elevated liver enzymes, rash, and diarrhea, with bone pain being the most common. This is consistent with other reports on G-CSF-related AEs [6,26,30,32]. Mild to moderate bone pain is one of the most frequently reported AEs. No new AEs were observed in our study, and the incidence of AEs did not differ significantly between the two groups. Furthermore, no infection-related deaths occurred following G-CSF administration in our study cohort, which might be related to the sample size. Mortality due to FN-related infections in adult and other pediatric studies is approximately 0.25–0.75% [7,8,9]. Although formal psychological or compliance assessments were not performed, clinical observation suggested that families appreciated the reduced injection frequency with the single-dose PEG-rhG-CSF regimen, which may have improved adherence and reduced treatment burden. This practical advantage aligns with the introduction regarding the burden of daily injections in pediatric patients.

### 4.1. Limitations

This study has several limitations. First, it was a single-center study with a relatively small sample size, particularly in subgroup analyses. Second, the open-label design may have introduced bias, although the primary outcome (FN incidence) was objectively defined. Third, the trial was registered at a hospital-based registry rather than an international public registry. Fourth, the study was not powered to detect differences in secondary outcomes such as hospitalization costs or transfusion requirements. Future multicenter, blinded, randomized controlled trials with larger cohorts are warranted to confirm these findings. Fifth, given the cycle-level independent randomization design, the potential for carry-over effects between chemotherapy cycles in the same patient cannot be fully excluded, although the cycle interval of at least 12 days may mitigate such effects. In addition, the sequence of G-CSF formulations received across cycles was not formally tested for its impact on outcomes, and we acknowledge that formal carry-over testing was not performed. Additionally, residual confounding related to chemotherapy intensity or treatment phase may remain despite the use of GEE and mixed-effects models to account for within-patient correlation. Sixth, the 2:1 allocation ratio was chosen to allow more patients to receive the investigational agent, but this imbalance may reduce statistical efficiency compared with 1:1 randomization for some secondary endpoints. Seventh, the sample size was insufficient to detect rare or clinically important differences in adverse events. Eighth, detailed data on prior FN episodes and other baseline risk factors were not collected in this study, which may limit the assessment of individual baseline risk.

### 4.2. Clinical Implications

Despite these limitations, our findings have important clinical implications. The single-dose administration of PEG-rhG-CSF offers a significant practical advantage over daily injections, potentially improving adherence, reducing treatment burden on children and families, and minimizing disruptions in therapy. This convenience factor, coupled with its comparable or superior efficacy, positions PEG-rhG-CSF as a valuable supportive care option for pediatric patients with hematological malignancies receiving myelosuppressive chemotherapy.

## 5. Conclusions

In this single-center, open-label trial, prophylactic single-dose PEG-rhG-CSF was associated with a lower incidence of FN compared to daily short-acting rhG-CSF. Safety profiles appeared broadly similar, but the sample size was insufficient to detect rare differences. These findings are hypothesis-generating and require confirmation in larger multicenter patient-level trials.

## Figures and Tables

**Table 1:** Comparison of Baseline Characteristics Between the Experimental and Control Groups.

Characteristic	Experimental Group	Control Group
**Demographics (n)**	53	26
Male [n (%)]	37 (69.8)	16 (61.5)
Age (years)	7.0 ± 3.8	6.7 ± 3.5
**Chemotherapy cycles (n)**	86	45
Disease Type n (%)		
ALL	19 (22.0)	9 (20.0)
AML	20 (23.2)	12 (26.7)
NHL	47 (54.7)	24 (53.3)
Chemotherapy phase n (%)		
Induction	14 (16.3)	6 (13.3)
Consolidation	66 (76.7)	36 (80.0)
Re-induction	6 (7.0)	3 (6.7)
Prior chemotherapy cycles	4.37 ± 3.28	4.87 ± 3.30

ALL: Acute Lymphoblastic Leukemia; AML: Acute Myeloid Leukemia; NHL: Non-Hodgkin’s Lymphoma.

**Table 2:** Comparison of Efficacy Between the Experimental and Control Groups.

Outcome Measure	Experimental Group (*n* = 86)	Control Group (*n* = 45)	OR/MD (95% CI)	*p*-Value
			OR *	
Neutropenia [n (%)]	73 (84.9)	44 (97.8)	0.26 (0.05–1.26)	0.095
FN [n (%)]	51 (59.3)	35 (77.7)	0.42 (0.18–0.97)	0.046
			MD **	
FN duration (days)	8.2 ± 8.4	8.2 ± 5.9	1.87 (−0.66–4.40)	0.96
RBC Transfusions (n)	0.7 ± 1.5	0.6 ± 1.0	−0.21 (−0.69–0.28)	0.43
Platelet Transfusions (n)	1.2 ± 1.1	1.2 ± 1.2	0.06 (−0.37–0.49)	0.97
Hospital Stay (days)	22.0 ± 10.8	21.7 ± 5.8	−0.43 (−5.02–4.16)	0.87
Hospitalization Costs (CNY)	46,301.7 ± 34,650.5	42,923.4 ± 17,151.5	−722 (−15,047–13,603)	0.49

* ORs were obtained from GEE analyses, with patient ID as the subject (cluster) variable to adjust for within-patient correlation. ** MDs were derived from linear mixed-effects models, with patient ID as a random intercept to account for within-patient correlation. FN: Febrile Neutropenia.

**Table 3:** Comparison of Efficacy Across Different Tumor Type Subgroups.

Subgroup/Outcome	Experimental Group	Control Group	OR (95% CI) *	*p*-Value *
AL (ALL + AML)	(*n* = 39)	(*n* = 21)		
Neutropenia [n (%)]	32 (82.1)	20 (95.2)	0.48 (0.08–2.77)	0.413
FN [n (%)]	24 (61.5)	15 (71.4)	0.64 (0.18–2.29)	0.492
NHL	(*n* = 47)	(*n* = 24)		
Neutropenia [n (%)]	41 (87.2)	24 (100)	/ **	/ **
FN [n (%)]	27 (57.4)	20 (83.3)	0.27 (0.08–0.89)	0.032

*: *p*-values and ORs were obtained from GEE analyses, with patient ID as the subject (cluster) variable to adjust for within-patient correlation. **: In the NHL subgroup, the incidence of neutropenia was 100% (24/24) in the control group; therefore, the GEE model did not converge due to complete separation, and only descriptive data are reported for this outcome; FN: Febrile Neutropenia; AL: Acute Leukemia; ALL: Acute Lymphoblastic Leukemia; AML: Acute Myeloid Leukemia; NHL: Non-Hodgkin’s Lymphoma.

## Data Availability

The datasets generated and/or analyzed during the current study are available from the corresponding authors upon reasonable request. National Medical Research Registration and Filing Information System (9450100201000RQ).

## Supplementary Materials

The following supporting information can be downloaded at: https://www.mdpi.com/article/10.3390/cancers18142214/s1. File S1: CONSORT checklist; File S2: Supplementary Methods; Table S1: Chemotherapy Regimens and Phases. Refs. [27,33,34] are cited in Supplementary Materials.
