# Supplementary material for: Prophylactic PEG-rhG-CSF Reduces Febrile Neutropenia in Pediatric Hematological Malignancies Compared with Daily rhG-CSF
Source: Cancers (Basel). 2026 Jul 9;18(14):2214. doi: 10.3390/cancers18142214 (PMC13406966; doi:10.3390/cancers18142214)
Supplement: Supplementary file 1 [file cancers-18-02214-s001.zip › cancers-4406888-File S2.pdf]

Supplementary Methods

1. Detailed Inclusion and Exclusion Criteria

Inclusion Criteria:

1. Age < 18 years, regardless of sex.
2. Diagnosis of pediatric hematological malignancy (leukemia or lymphoma) confirmed by bone marrow pathology or cytology.
3. Scheduled to receive prophylactic PEG-rhG-CSF or rhG-CSF after chemotherapy, with an interval of at least 12 days between chemotherapy cycles.
4. Adequate liver and kidney function: pre-chemotherapy total bilirubin, alanine aminotransferase (ALT), and aspartate aminotransferase (AST) all < 2.5 times the upper limit of normal (ULN); serum creatinine < 2 times ULN.
5. Good mental status and consciousness; legal guardian provided written informed consent.

Exclusion Criteria:

1. Severe dysfunction of vital organs.
2. Use of other investigational drugs or participation in other clinical trials within 4 weeks prior to enrollment.
3. Known allergies to PEG-rhG-CSF, rhG-CSF, or other preparations/proteins expressed in *E. coli*.
4. Any other condition deemed by the investigator as unsuitable for participation.

2. Detailed Withdrawal Criteria

Patients were withdrawn from the study if any of the following occurred:

1. Use of prohibited medications (any G-CSF other than the study drug) during the trial.
2. Other serious protocol violations.

Study Dropout: Any subject who signed informed consent and passed screening had the right to withdraw from the clinical trial at any time. Dropout criteria included:

1. Patients unwilling or unable to continue the trial, requesting withdrawal from

the investigator; or withdrawal of informed consent at any time.

2. Patients lost to follow-up who ceased receiving medication and examinations without explicitly withdrawing.
3. Patients with worsening complications or comorbidities judged by the investigator as difficult to continue medication.

3. Randomization Method Details

Randomization was performed at the chemotherapy-cycle level. For each eligible chemotherapy cycle, patients were randomly assigned in a 2:1 ratio to receive either PEG-rhG-CSF or short-acting rhG-CSF. A single patient could contribute multiple chemotherapy cycles over the course of treatment, with each cycle independently randomized.

Prior to study initiation, a computer-generated random number list was created to assign participants in a 2:1 ratio to the experimental group (PEG-rhG-CSF) or control group (short-acting rhG-CSF). Sequentially numbered, opaque, sealed envelopes containing the group assignments were prepared. Each envelope was numbered externally without revealing the group assignment. The contents remained confidential and were accessible only to the investigating researcher during study implementation.

4. Sample Size Calculation

A 2:1 randomization ratio was selected to allow more patients to receive the investigational long-acting G-CSF while maintaining a valid control group, consistent with the sample size calculation and the exploratory nature of this trial.

The sample size was estimated based on the primary endpoint, which was the incidence of FN. For the acute leukemia subgroup, a superiority design was adopted. Based on a previously reported FN rate of 62.5% in the control group receiving short-acting rhG-CSF from our institution's earlier study [1], and assuming that PEG-rhG-CSF would reduce the FN rate by at least 20% to 42.5% [2], with a one-sided alpha level of 0.05 and a statistical power of 80%, the required sample size was determined to be 43 cycles in the PEG-rhG-CSF group and 22 cycles in the control group, with a 2:1 randomization ratio.

For the lymphoma subgroup, the calculation was based on a control FN rate of 50.8% from the same institutional study. Assuming a similar reduction of 20% to 30.8% with PEG-rhG-CSF, and using the same statistical parameters, the estimated sample size was 49 cycles for the PEG-rhG-CSF group and 25 cycles for the control group.

The total planned sample size was approximately 140 cycles, accounting for the fact that individual patients might contribute multiple chemotherapy cycles. The actual enrollment of 131 cycles closely matched the planned target, confirming that the study was adequately powered to address the primary objective.

5. Study Drugs Details

1. **Experimental Group:** Pegylated recombinant human granulocyte colony-stimulating factor injection (PEG-rhG-CSF) [Specification: 3.0 mg (1 mL)/vial, CSPC Pharmaceutical Group Limited]. Administration: Subcutaneous injection at 100 μg/kg, once per chemotherapy cycle, administered 24–72 hours after chemotherapy completion.

2. **Control Group:** Short-acting recombinant human granulocyte colony-stimulating factor (rhG-CSF) (brand not restricted). Administration: Subcutaneous injection at 5 μg/kg/day, starting 24–72 hours after chemotherapy completion, continued until absolute neutrophil count (ANC) exceeded the nadir and remained $> 0.5 \times 10^9$/L for two consecutive days.

3. **Concomitant Medications:** During chemotherapy, patients received symptomatic treatments such as antiemetics as clinically indicated. Prophylactic antibiotics were not administered routinely. Antibiotic therapy was initiated only when patients developed grade 4 neutropenia, febrile neutropenia with documented infection, or fever ≥38°C that could not be ruled out as infectious in origin.

6. Chemotherapy Regimens Details

The specific chemotherapy regimens and phases used in this study are summarized in **Supplementary Table S1**. All regimens were administered according to the respective protocols: CCCG-ALL-2020 for acute lymphoblastic leukemia (ALL), CALSIII-AML18 for acute myeloid leukemia (AML), and CNCL-NHL-2017 (subdivided for lymphoblastic lymphoma and mature B-cell lymphoma) for non-Hodgkin lymphoma (NHL). Prophylactic PEG-rhG-CSF was used only when the interval between two chemotherapy cycles was at least 12 days. Drug doses followed the standard protocols.

References

1. Fu Y, Wang HS, Zhai XW, et al. Prophylactic recombinant human granulocyte/granulocyte-macrophage colony-stimulating factor in children with hematologic malignancies after chemotherapy. Tumor. 2017;37(5):504-510.

2. Spunt SL, Irving H, Frost J, et al. Phase II, randomized, open-label study of pegfilgrastim-supported VDC/IE chemotherapy in pediatric sarcoma patients. J Clin Oncol. 2010;28(8):1329-1336.
