# Supplementary material for: Prophylactic PEG-rhG-CSF Reduces Febrile Neutropenia in Pediatric Hematological Malignancies Compared with Daily rhG-CSF
Source: Cancers (Basel). 2026 Jul 9;18(14):2214. doi: 10.3390/cancers18142214 (PMC13406966; doi:10.3390/cancers18142214)
Supplement: Supplementary file 1 [file cancers-18-02214-s001.zip › cancers-4406888-Table S1.pdf]

Supplementary Table S1. Chemotherapy Regimens and Phases

Chemotherapy Regimen	Chemotherapy Phase	Chemotherapeutic Drugs
CCCG-ALL-2020	CAT	CTX、Ara-C、6-MP
	CAT+	CTX、Ara-C、6-MP、VCR、PEG-ASP
	Reinduction	DEX、VCR、Ara-C、PEG-ASP
CALSIII-AML-18	MAG	MIT/IDA、Ara-C、G-CSF
	DAE	DNR、Ara-C、VP16
	HA、EA、LA	HHT/VP16/L-ASP、Ara-C
	FLAG	FLU、Ara-C、DEX、G-CSF
CNCL-NHL-2017-LBL	CAM	CTX、Ara-C、6-MP
	HR1	DEX、VCR、MTX、CTX、Ara-C、PEG-ASP
	HR2	DEX、VDS、MTX、IFO、DNR、PEG-ASP
	HR3	DEX、Ara-C、VP16、PEG-ASP
CNCL-NHL-2017- Mature B	COPADM3/5	VCR、Pred、CTX、DNR、MTX、FH4Ca
	CYVE1/2	Ara-C、VP16、（MTX）
	$M_5$1	VCR、Pred、MTX、CF、CTX、E-ADR
	M2、M4	Ara-C、VP16
	M3	VCR、Pred、CTX、E-ADR

Ara-C: Cytarabine; CF: Calcium Folinate; CTX: Cyclophosphamide; DNR: Doxorubicin; DEX: Dexamethasone；E-ADR: Epirubicin; FH4Ca: Calcium Leucovorin; FLU: Fludarabine; G-CSF: Granulocyte Colony-Stimulating Factor; HHT: Homharringtonine; IDA: Idarubicin; IFO: Ifosfamide; MIT: Mitoxantrone; MTX: Methotrexate；PEG-ASP: Pegylated Asparaginase; Pred: Prednisone; VCR: Vincristine; VDS: Vinblastine; VP16: Etoposide.

Note: This supplementary material provides additional methodological details that could not be included in the main manuscript due to space limitations. The content has been reviewed and approved by all authors.
